# Phase I study of QLNC120, a novel EGFR and HER2 kinase inhibitor, in pre-treated patients with HER2-overexpressing advanced breast cancer

**DOI:** 10.18632/oncotarget.13581

**Published:** 2016-11-25

**Authors:** Tongtong Zhang, Qing Li, Shanshan Chen, Yang Luo, Ying Fan, Binghe Xu

**Affiliations:** ^1^ Department of Medical Oncology, National Cancer Center/Cancer Hospital, Chinese Academy of Medical Sciences and Peking Union Medical College, Beijing 100021, China

**Keywords:** QLNC120, tyrosine kinase inhibitor, Phase I, HRAS

## Abstract

This study evaluated the dose-limiting toxicities (DLT), maximum tolerated dose (MTD), pharmacokinetic profile, and preliminary antitumor activity of QLNC120, an inhibitor of epidermal growth factor receptor (EGFR) and human epidermal growth factor receptor 2 (HER2), in HER2 overexpressing advanced breast cancer patients. In addition, the prognostic biomarkers of QLNC120 were investigated. QLNC120 was administered as a single dose, followed by 7 days observation, and then once daily consecutively. Scheduled dose escalation was 450mg, 750mg, 1000mg and 1250mg. For pharmacokinetic analysis, blood samples were collected after the single dose and after the first 7 days of continuous administration. Tissue samples were collected for biomarker analysis. Twenty-four heavily treated HER2 overexpressing advanced breast cancer patients were enrolled. No DLT was observed. MTD was not found. QLNC120 and its active metabolite-lapatinib exposure did not increase in a dose-dependent manner ranging from 450 to 1250mg QLNC120. From 450 to 1250mg QLNC120, the exposure of combination of QLNC120 and its active metabolite-lapatinib was equal to or greater than the exposure of 1250mg lapatinib. Common QLNC120-related toxicities included rash, diarrhea, oral mucositis, hematuria and white blood cell decrease. Seven of twenty-two evaluable patients achieved partial response (PR) or stable disease (SD)≥24 weeks. In biomarker analysis, nine of fifteen patients (60%) had a mutation in HRAS exon 1. Patients with HRAS mutation achieved longer progression free survival(PFS) (24.9 vs 12.9 weeks, p=0.023, HR=0.291). QLNC120 is well-tolerated and safe with encouraging antitumor activity in HER2 overexpressing advanced breast cancer. HRAS mutation was associated with the anti-tumor activity of QLNC120. (Trial registration: NCT01931943, http://ClinicalTrials.gov/show/NCT01931943)

## INTRODUCTION

The epidermal growth factor (ErbB) family of membrane receptor tyrosine kinases comprises EGFR, HER2, HER3 and HER4 [1]. Numerous ligands have been identified that interact with the entire HER family. HER2 is overexpressed in 20% to 25% of breast cancers, and is associated with a poor prognosis [2]. Some small-molecule tyrosine kinase inhibitors (TKIs) (e.g., lapatinib, neratinib) and antibodies (e.g., trastuzumab, trastuzumab-DM1, pertuzumab) targeting the erbB family have been developed to treat HER2 positive breast cancer. Despite these new therapeutic options, disease progression of HER2-directed therapy is experienced by most patients, and new strategies are needed to delay or overcome the onset of tumor progression.

Preclinical studies revealed that QLNC120 has high inhibitory activity to HER2 and EGFR with IC50 values of 2.3 nmol/L and 4.0±3.2 nmol/L respectively by using ELISA method. QLNC120 did not demonstrate an effect on the pathways of c-Kit, KDR, PDGFRβ, c-Src and c-Met. In BT474, SK-BR-3 and NCI-N87 cell lines with HER2 or EGFR overexpression, QLNC120 produced highly inhibitory activities with IC50 values of 35.4 nM, 56.0 nM and 134 nM respectively. For doses ranging from 50 to 200 mg/kg, QLNC120 showed modest inhibitory activity in HER2 over-expressed BT-474, SK-OV-3, NCI-N87 xenograft model. The results of *in vivo* research demonstrated that the anti-tumor activity of QLNC120 was dose-dependent. In the NCI-N87 xenograft model, the exposure dose of 100 mg/kg of QLNC120 was lower compared to 200 mg/kg of lapatinib. However, the exposure of active agents in tumor tissue and the anti-tumor activity were similar (inhibitory rate 71% vs. 67%). When compared to the anti-tumor activity of Lapatinib *in vivo*, QLNC120 efficacy was superior with less toxicity. Additionally, the inhibitory effect of QLNC120 was less during *in vitro* evaluation of cardiac toxicity. In acute toxicity research, the MTDs of Sprague Dawley (SD) rats and Beagles were >2000 mg/kg and MTD>1000 mg/kg respectively. The long term toxicity test results of QLNC120 in SD rats and Beagles were NOAEL=75 mg/kg and NOAEL>15 mg/kg respectively. The pharmacokinetic parameters of QLNC120 were tested in SD rats, Beagles and *in vitro* models. The absolute bioavailability of QLNC120 ranged from 30% to 48%. After 5 days of consecutive oral administration of QLNC120, the serum QLNC120 reached steady state. In SD rats and Beagles, QLNC120 was observed to be biotransformed to lapatinib. The Lapatinib AUC_0-t_/ QLNC120 AUC_0-t_ were 51% and 44% in female rat and Beagles respectively. The T_1/2_ of QLNC120 and its metabolite, lapatinib, ranged from 2.8h to 9h in SD rats and Beagles. The maximum concentration of QLNC120 and lapatinib was observed at approximately 2.5-9 hours post dose in our *in vivo* model. Both QLNC120 and lapatinib were observed to be highly plasma protein bound (>90%). QLNC120 was observed to be mainly biotransformed into lapatinib (73%), by CYP3A4*in vivo* and in liver microsomes. After a single oral dose of 60 mg/kg QLNC120 in SD rats, the excretion of the parent drug and its metabolites in feces, bile and urine was 58.4%, 6.55% and 0.1% of the dose, respectively.

Ras is one of the more frequently mutated oncogenes in many human cancers (30%) [3]. The frequency of Ras seen in breast cancer is less than 5% [4]. The Ras protein is involved in many cellular signaling pathways including cell growth, migration, cytoskeletal integrity, survival and differentiation [5, 6]. As an oncogene, Ras can be activated either by gene amplification and/or mutation [7]. Point mutations are the most frequently observed product of Ras gene activation [8]. The Ras gene family consists of 3 members: HRAS, KRAS and NRAS [9]. Among these three members, the mutation or aberrant expression of HRAS is most frequent in breast cancer [7, 9]. HRAS is a small G protein in the Ras subfamily of the Ras superfamily of small GTPases [10]. Raf activates MAPK (mitogen-activated protein kinase), PI3K (Phosphoinositide 3-kinase) and RalGDS (Ras-like guanine nucleotide-dissociation stimulator). These were identified as the three main downstream signal pathways of HRAS [11–13]. However, the relationship between HRAS mutation with breast cancer treatment is still unclear.

## RESULTS

### Patient characteristics

Twenty four patients were enrolled between 11 Apr 2013 and 19 Aug 2014. All patients completed the single dose tolerability trial, multiple dose tolerability trial, entered into continuous treatment phase, and were evaluable for DLT assessment. Patient characteristics are shown in Table 1. The mean age, ECOG performance status, previous chemotherapy regimens and trastuzumab treatment are listed respectively (Table 1). All patients had advanced HER2-overexpressing breast cancer. They were all heavily pretreated and received systemic chemotherapy. Sixteen patients received trastuzumab before being enrolled in this trial. Six patients received QLNC120 starting doses of 450, 750, 1000, and 1250 mg/d.

**Table 1:** Patient character

	Dose group			
	450mg(n=6)	750mg(n=6)	1000mg(n=6)	1250mg(n=6)
Age, mean (range)	51.3±9.00	54.8±3.13	46.3±7.94	55.5±10.13
ECOG performance status				
0	4	6	5	6
1	2	0	1	0
2	0	0	0	0
previous chemotherapy regimens				
0	2	1	0	0
1-3	3	5	3	5
3-6	1		2	1
>6	0	0	1	0
endocrine therapy				
Yes/No	3/3	1/5	6/0	2/4
trastuzumab treatment	4	4	5	3

### Sequence of dose levels studied and DLTs

Six patients were enrolled in dose level 1 (QLNC120 450 mg). Two patients experienced drug-related AEs (grade 1 serum creatine phosphokinase increase, rash, neutropenia) and one patient experienced a severe adverse event (grade 2 thromboembolic event) unrelated to QLNC120. These events were not considered DLTs, therefore investigators decided to explore a higher dose cohort.

At dose level 2 (QLNC120 750 mg), six patients were enrolled. Three patients experienced drug-related AEs (grade 1 hiccups, diarrhea, gastroesophageal reflux disease, leukopenia, nausea, vomiting, neutropenia, elevated alanine amino transferase, grade 2 oral mucositis, and toothache). Since DLTs were not experienced by any patients at this dose level, investigators decided to increase to the next dose level in the next dose cohort.

Six patients were enrolled in dose level 3 (QLNC120 1000 mg). This dose level was well tolerated. Due to absence of DLTs at this dose level and the previous dose level, investigators recommended increasing the dose level to QLNC120 1250 mg.

Six patients were enrolled in dose level 4 (QLNC120 1250 mg). All patients were well tolerated and no patients experienced DLTs. Since all planned dose levels were completed and dose level 4 reached a clinically effective dose, investigators decided not to explore a higher dose cohort.

### Safety and tolerability

All patients were evaluated for toxicity. Each patient experienced at least one AE. The main drug-related AEs were: elevated serum creatine phosphokinase, rash, neutropenia, oral mucositis, hematuria and leukopenia. The frequency of drug-related adverse events in each group was similar. All treatment related AEs are shown in Table 2. In total, nine patients experienced grade 2 AEs. No patients experienced grade 3 drug related AEs. Two grade 2 drug related AEs occurred in the 450 mg QLNC120 cohort (serum creatine phosphokinase increase and fatigue). One patient in the 750 mg QLNC120 cohort experienced a grade 2 drug related AE (oral mucositis). Four grade 2 drug related AEs occurred in the 1000 mg QLNC120 cohort (Serum creatine phosphokinase increase, elevated ALT, rash and bronchial infection). Two grade 2 drug related AEs happened in the 1250 mg QLNC120 cohort (GGT increased and diarrhea). In total, three cases of grade 3 AEs (Pain in the extremities, hypertriglyceridemia, elevated GGT) were observed and all considered to be unrelated to QLNC120. In this study, no patients experienced grade 4 AEs. (Table 2)

**Table 2:** Treatment relevant adverse events

		Dose group			
AE type	Grade	450mg(n=6)	750mg(n=6)	1000mg(n=6)	1250mg(n=6)
Serum creatine phosphokinase increase	Grade1	2/6	4/6	1/6	1/6
	Grade2	1/6	0/6	1/6	0/6
Rash	Grade1	2/6	3/6	0/6	2/6
	Grade2	0/6	0/6	1/6	0/6
Fatigue	Grade1	2/6	3/6	2/6	2/6
	Grade2	1/6	0/6	0/6	0/6
Diarrhea	Grade1	3/6	3/6	2/6	1/6
	Grade2	0/6	0/6	0/6	1/6
neutrophil count decrease	Grade1	2/6	1/6	2/6	0/6
	Grade2	0/6	0/6	0/6	0/6
oral mucositis	Grade1	0/6	0/6	1/6	2/6
	Grade2	0/6	1/6	0/6	0/6
hematuria	Grade1	1/6	2/6	0/6	0/6
	Grade2	0/6	0/6	0/6	0/6
white blood cell decrease	Grade1	0/6	2/6	3/6	0/6
	Grade2	0/6	0/6	0/6	0/6
alanine aminotransferase increase	Grade1	3/6	4/6	1/6	1/6
	Grade2	0/6	0/6	1/6	0/6
γ-glutamyltransferase increase	Grade1	0/6	0/6	0/6	0/6
	Grade2	0/6	0/6	0/6	1/6
bronchial infection	Grade1	0/6	0/6	0/6	0/6
	Grade2	0/6	0/6	1/6	0/6

### Pharmacokinetics

**Figure 1:**
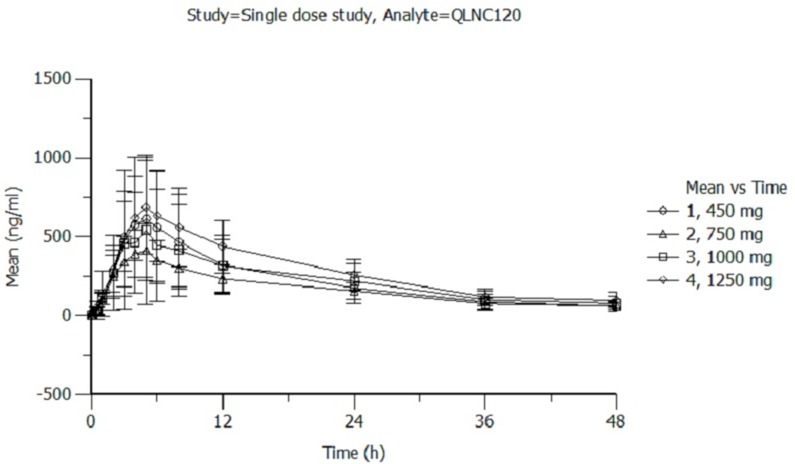

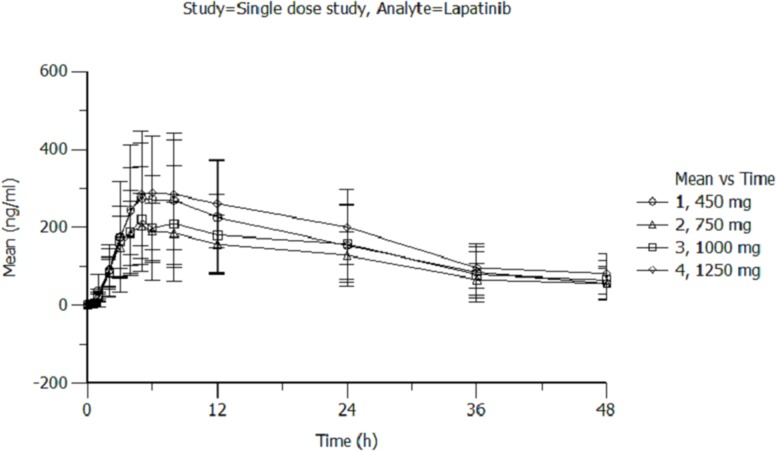
**A**. The pharmacokinetics of single dose of QLNC120 and its active metabolites lapatinib. **B**. The pharmacokinetics of single dose of QLNC120 and its active metabolites lapatinib.

All patients enrolled in the pharmacokinetic phase completed both single dose and multiple-dose pharmacokinetic study. The pharmacokinetic parameters for single dose of QLNC120 are shown in Figure 1A. After treatment with a single dose of QLNC120 ranging from 450-1250 mg, absorption was relatively slow, with a median t_max_ of 4-6.5 hours. Furthermore, C_max_ did not relatively change, while the AUC increased less than dose proportional to dose. The AUC of lapatinib (Figure 1B) accounted for 60% of AUC of parent drug. On day 1, following a single oral dose range of 450-1250 mg, QLNC120 exposure did not increase in a dose-dependent manner. The terminal half-life of QLNC120 and its active metabolite (lapatinib) ranged from 12.4-15.9h and 18.7-23.0h respectively.

**Figure 2:**
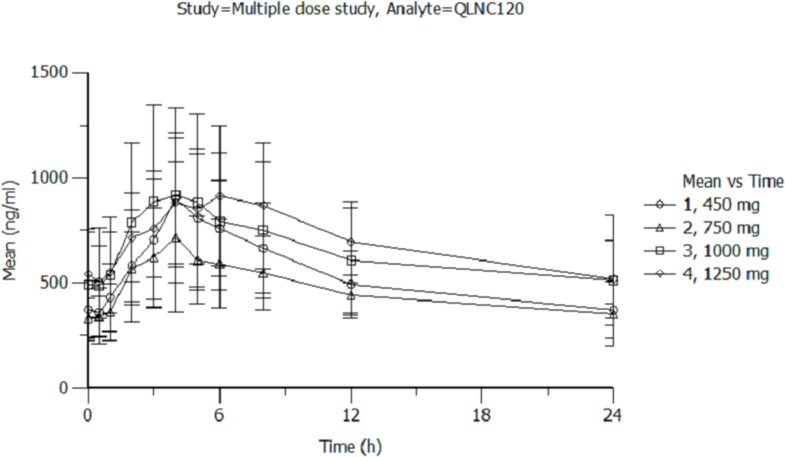

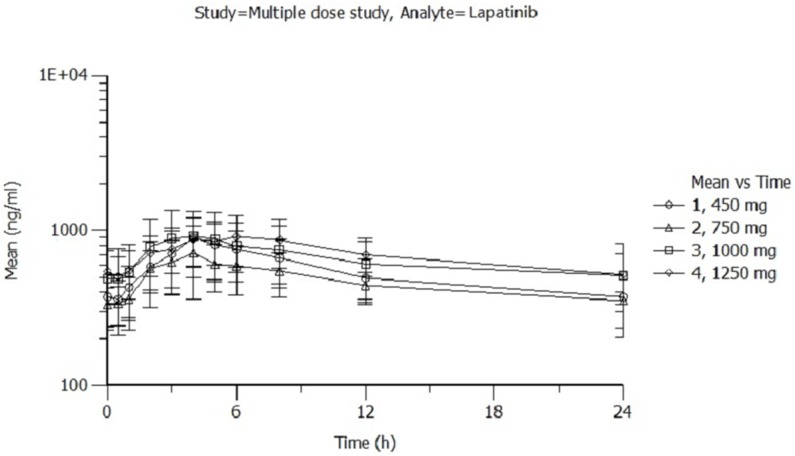
**A**. The pharmacokinetics of multiple doses of QLNC120 and its active metabolites lapatinib. **B**. The pharmacokinetics of multiple doses of QLNC120 and its active metabolites lapatinib.

For multiple-dose pharmacokinetic study, C_max_ and AUC_0–24h_ increased less than proportional to dose for dose levels from 450 mg to 1250 mg. Additionally, C_max_ was effectively unchanged and AUC increased, but less than the dose increasing ratio. For the same respective doses on day 7 at steady state, the mean C_max_ of QLNC120 (Figure 2A) ranged from 0.729-1.02 ng/mL, the mean C_max_ of lapatinib (Figure 2B) ranged from 0.38-0.515 ng/mL, and the AUC_ss_ of QLNC120 ranged from 11.2-16.7 h*ng/mL while AUC_ss_ of lapatinib ranged from 7.07-8.64 h*ng/mL. The accumulation ratio was less than 3 based on the exposure of QLNC120 and lapatinib (R, AUC_ss_ on study day 7 to AUC_0-24h_ on study day 1). These results suggest that there was no serious accumulation of QLNC120 and its active metabolite, lapatinib, after repeated daily administration to patients.

### Clinical activity

Twenty-two patients were evaluable for response. Two patients were excluded for clinical efficacy analysis (1 enrolled ineligible patient, 1 patient received radiotherapy with 4 weeks of enrollment). The overall clinical benefit rate (CBR) was 33.3% (7 of 22). The best response was a PR in three patients and four of seven patients had SD for ≥ 6 months. The median PFS was 15 weeks. The percentage of patients whose disease was progression free at 8, 16, 24 weeks were 68.2%, 50.0% and 31.8% respectively. 15 of 22 trastuzumab-refractory patients, 7 of them achieved PR or had SD for ≥ 6 months. The CBR of trastuzumab resistant patients was 46.7%.

At dose level 1, one patient achieved PR and 2 patients maintained SD >6 months.

At dose level 2, one patient achieved PR and 1 patient maintained SD >6 months.

At dose level 3, one patient achieved PR and 1 patient maintained SD >6 months.

At dose level 4, no patients achieved PR or maintained SD more than 6 months.

### Biomarker analysis

**Figure 3:**
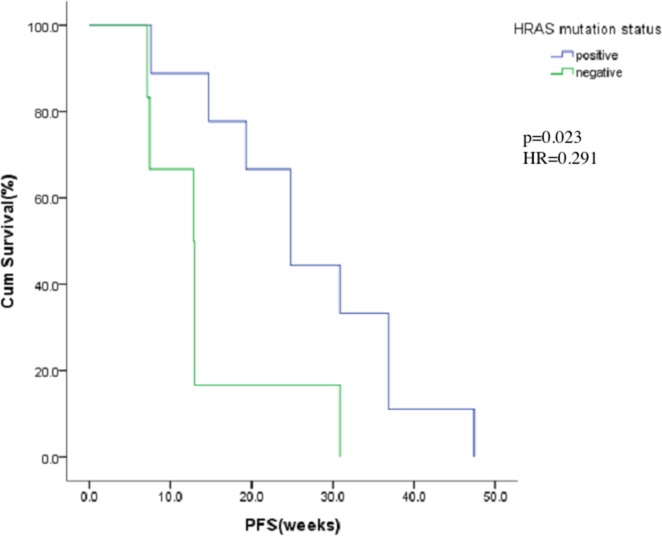
Kaplan-Meier estimates of progress-free for HRAS mutation positive or negative

Fifteen patients signed informed consent for this additional biomarker study. A mutation in HRAS exon 1 was observed in 9 (60%) of 15 patients. PFS of patients with and without HRAS mutation were 24.9 weeks and 12.9 weeks respectively. Among these 15 patients, those with HRAS mutation achieved longer PFS period (*p*=0.023, HR=0.291). The relationship between HRAS mutation and the PFS in these 15 patients is shown in Figure 3.

## DISCUSSION

In this phase I study, QLNC120 was well tolerated at doses up to 1250 mg daily with clinical activity at doses ranging from 450 mg to 1000 mg in heavily pretreated patients with HER2 overexpressing advanced breast cancer. No DLTs were observed in all 4 dose groups ranging from 450 mg to 1250 mg. All scheduled dose groups were completed and higher doses were not explored.

QLNC120 was found to be safe and well tolerated. There was no correlation between dose level and severity or frequency of drug related AEs. The most common related adverse events were rash, oral mucositis, diarrhea and elevated serum creatine phosphokinase. All of these drug-related adverse events were easy to manage and well tolerated. The frequency of diarrhea and rash was similar between patients given QLNC120 or lapatinib. However, we saw less severity in the QLNC120 group since we did not observe occurrences of these two drug-related adverse events greater than grade 2 [14, 15]. Commonly observed adverse events for QLNC120, (elevated serum creatine phosphokinase and oral mucositis), are less frequently found in patients given lapatinib [16]. We found no apparent cardiac toxicity as there were no reports of cardiac adverse events (similarly to laptinib).

Although the PK information of all enrolled patients in single and multiple dose phases were collected, we were not able to fully evaluate the PK profiles for QLNC120. According to the regulations and guidelines for phase I studies in China [17], PK studies were conducted in at least eight patients per dose cohort. PK analysis showed that with increasing exposure of QLNC120, the AUC of QLNC120 and lapatinib did not apparently change. All PK parameters displayed moderate to high variability similar to other orally administered TKIs (eg. Lapatinib [18], erlotinib [19], gefitinib [20], and afatinib [21]). The exposure of combination QLNC120 and lapatinib (ranging from 450-1250 mg), was equal to or greater than the exposure of 1250 mg lapatinib. At all dose levels, the AUC of lapatinib in serum was about 60% of the AUC of the parent drug indicating that biotransformation was not statured.

Clinical efficacy results demonstrated that heavily pretreated advanced breast cancer patients can benefit from QLNC120. Clinical responses were observed for all dose levels of QLNC120. Three patients achieved a confirmed PR and 4 (13.6%) patients demonstrated SD ≥ 6 months. The CBR of 22 evaluable patients was 33.3%. Notably, this study observed the CBR of trastuzumab-refractory patients to be 46.7%, higher than that of lapatinib (25%) [22]. Consequently, trastuzumab-refractory patients may benefit from QLNC120. The median PFS was 15 weeks, longer than that of lapatinib (8.1 weeks). In addition, the percentage of patients whose disease was progression free at 24 weeks was 31.8%, compared to 13% observed in lapatinib monotherapy regimen [22]. However, due to the limited patient pool, the anti-tumor activity of QLNC120 in advanced breast cancer must be further explored.

Our study also demonstrated that patients with HRAS mutation achieved better PFS (24.9 vs. 12.9 weeks, *p*=0.023, HR=0.291). Many growth factor receptors such as EGFR [23, 24] are located in the upstream signaling pathways of RAS. RAS mutation is one of the most important mechanisms of EGFR TKI resistance [25, 26]. However, the prognostic value of HRAS in QLNC120 needs to be further investigated for accurate treatment. Additionally, due to limited patient numbers and lack of functional research on the relationship between HRAS and QLNC120, the exact mechanism remains to be elucidated.

In conclusion, oral QLNC120 is well-tolerated with encouraging antitumor activity in advanced breast cancer. The MTD of QLNC120 was not found in this study. HRAS mutation was associated with the anti-tumor activity of QLNC120.

## MATERIALS AND METHODS

### Patients

Eligible women were 18 to 65 years old with ECOG PS of 0-1, and histologically/cytologically confirmed advanced breast cancer. As determined by a local laboratory, HER2 overexpression was identified as IHC 3+ or fluorescence in situ hybridization (FISH) positive. Prior treatment with trastuzumab was permitted but not required. Additional eligibility criteria are as follows: At least one measurable disease site defined by Response Evaluation Criteria in Solid Tumors (RECIST v.1.1), life expectancy of at least 3 months and adequate hematology (white blood count of ≥3.5×10^9^/L, absolute neutrophil count of ≥1.5×10^9^/L, platelet count of ≥100×10^9^/L, hemoglobin of≥90 g/L), adequate hepatic function (serum bilirubin≤1.5 times upper limit of normal (ULN)), aspartate aminotransferase and alanine aminotransferase ≤1.5 × ULN, adequate renal function (creatinine and urea nitrogen≤1.5 × ULN), and adequate cardiac function (normal electrocardiography (ECG) and baseline left ventricular ejection fraction (LVEF) higher than 50%). Patients must be able to swallow and have normal gastrointestinal function. Patients must be recovered from any previous treatments. The interval must be more than six weeks since administration of nitroso or mitomycin. The interval of last radiotherapy treatment, other cytotoxic drugs or surgery should be more than 4 weeks. Patients should agree to take contraceptives during the study and for 6 months after the study (such as an intrauterine device [IUD], contraceptive drugs or condoms); Seven days before entering the study, serum or human chorionic gonadotropin should be negative, and must be in the non-lactation period. Exclusion criteria for this study are as follows: patients currently receiving small molecule targeted drug therapy of inhibition of HER-2 or EGFR, uncontrolled or significant cardiovascular disease, a left ventricular ejection fraction (LVEF) <45%, known interstitial lung disease or active brain metastases. Any patients who are pregnant or breast feeding, any clinically significant gastrointestinal abnormalities that can influence oral administration, patients with a history of symptomatic brain metastases, active serious infection, uncontrolled large pleural effusion and ascites, requirement for the therapeutic drugs prolonging QT interval (such as anti-arrhythmia drugs), any medical history of small molecule targeted drug therapy of inhibition of HER-2 or EGFR.

### Study design and treatment

**Figure 4:**
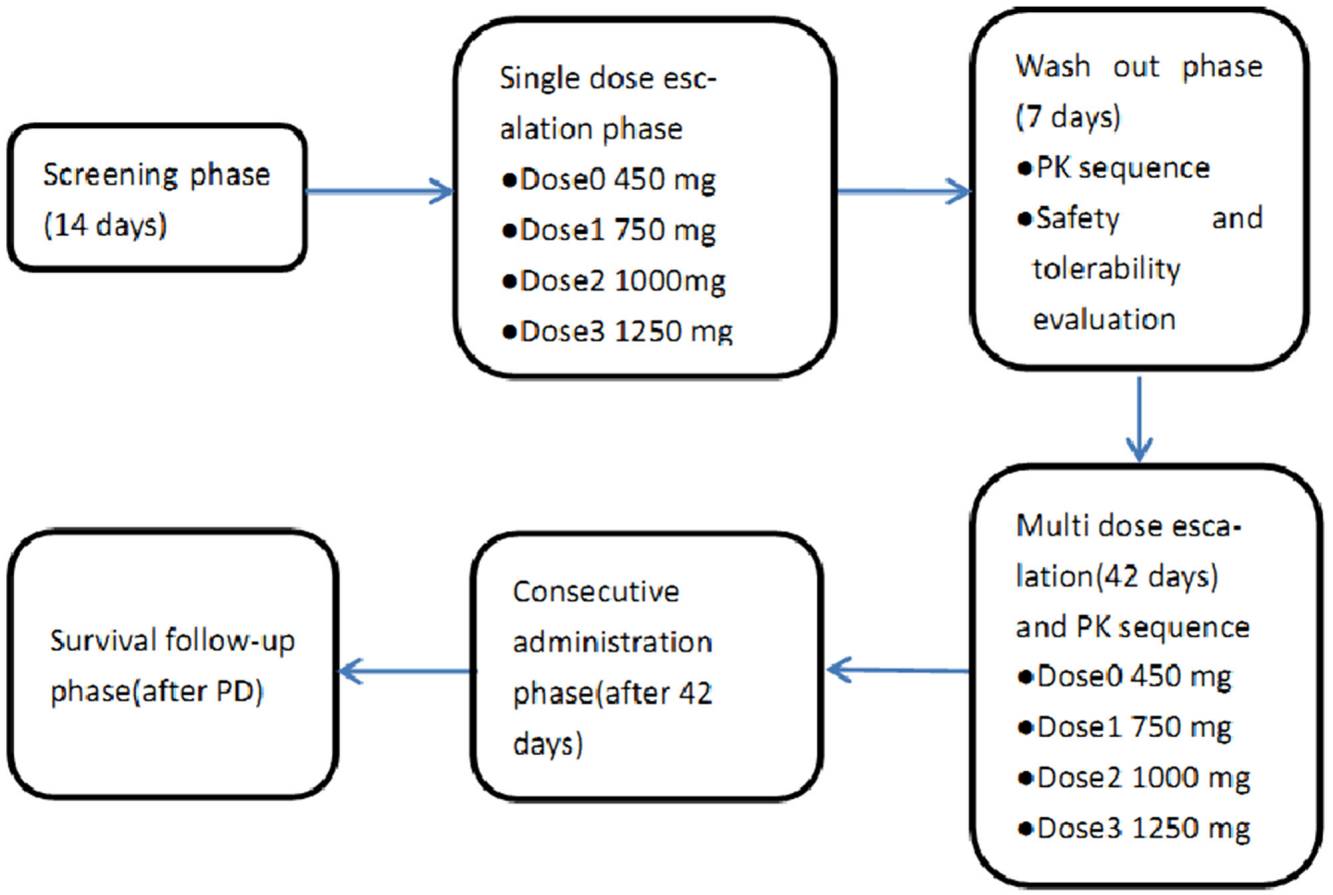
Study design

This was a Phase I, open-label, dose-escalation study (Trial registration ID: NCT01931943, http://ClinicalTrials.gov/show/NCT01931943) to evaluate QLNC120 in women with HER2 overexpressing advanced breast cancer. The primary aim of this study was to determine the safety and tolerability by measuring DLTs and MTD. The secondary objective were to determine the pharmacokinetics (PK) of QLNC120 following single and multiple dosing, and to evaluate the antitumor activity. Dose escalation followed a modified Fibonacci scheme plus 4 cohorts with 6 patients in each cohort. The planned dose-escalation schedule was 450 mg, 750 mg, 1000 mg, 1250 mg QLNC120 daily with food. Three patients in the first dose-escalation cohort were given a single dose of 450 mg QLNC120 and monitored 7 days for toxicity. If no DLT was observed, patients were entered into the next consecutive dose phase and received 450 mg QLNC120 daily starting on day 8. If no DLT was observed for 21 days, new patients can be enrolled in next dose level (750 mg QLNC120). Dose escalation continued until DLT was observed or a maximum dose level (1250 mg QLNC120) was reached. If one of three patients experienced a DLT at a particular dose level, an additional three patients were entered at that level. If no DLT was observed in the maximum dose group (1250mg), investigators and sponsors made a decision whether a higher dose should be explored or not (Figure 4). PK extension studies were performed to enroll additional patients up to at least 6 patients per cohort in each of the 4 dose levels for further evaluation of PK profiles. If two or more patients experienced DLTs (among the 3-6 patients for each cohort), dose escalation was terminated and the prior dose level was defined as the MTD.

### Study conduct

This study was conducted at a single site in China and was in compliance with the requirements of the Declaration of Helsinki, the International Conference on Harmonization Good Clinical Practice guidelines and relevant local guidelines. The protocol, informed consent and other trial-relevant documentations were reviewed and approved by the Institutional Review Board at Cancer Hospital, Chinese Academy of Medical Sciences. Written informed consent was obtained before participation for each patient.

### Safety and tolerability assessment

Safety and tolerability were evaluated and assessed by investigators according to the type and frequency of DLTs as referenced in Common Terminology Criteria for Adverse Events (CTC-AE) version 4.03 until day 21 of the first cycle of continuous dosing of QLNC120. DLTs were defined as follows: non-hematologic toxicity ≥2 cardiac dysfunction (LVEF decrease), renal function abnormality (creatinine) and other non-hematologic toxicity ≥3 (except alopecia, elevated alkaline phosphatase [ALP] and fever caused by a definitive reason such as a tumor). Hematologic toxicity: grade 4 neutropenia or febrile neutropenia, Grade 4 thrombocytopenia or grade 3 thrombocytopenia with clinical significance, Grade 4 anemia.

### Response assessment

Radiologic tumor assessments were conducted at baseline and every two cycles thereafter according to Response Evaluation Criteria In Solid Tumors guideline 1.1(RECIST 1.1) during study treatment. The primary efficacy parameter assessed in this trial was the clinical benefit rate (CBR), and CBR was defined as (CR+PR+SD>6 months)/all patients×100%.

### Pharmacokinetic analysis

All 24 patients receiving QLNC120 were evaluable for both single and multiple dose pharmacokinetic analysis. Patients received a single dose of investigational product, followed by a 7 day washout period and ended with a consecutive daily dose period. For each single dose period, blood samples for pharmacokinetic profiling of QLNC120 were collected at 0 (predose), 0.5, 1, 2, 3, 4, 5, 6, 8, 12, 24, 36 and 48 hrs post dosing. For the consecutive daily dose period, PK samples were obtained at 0 (predose), 0.5, 1, 2, 3, 4, 5, 6, 8, 12, 24, 120, 144 and 168 hrs postdosing. A same PK sampling schedule was applied for the PK extension phase.

Plasma concentrations of QLNC120 and its active metabolite (lapatinib) were measured using a validated liquid chromatography/tandem mass spectrometry method (LC-MS/MS). In total, 20 μl plasma was used for the bioanalysis. The range of QLNC120 and lapatinib was linear from 3 to 1000 ng/ml [lower limit of quantitation (LLQ) was 3 ng/ml]. The mean intraday variability (coefficient of variation) of QLNC120 and lapatinib quality control (QC) samples were V3.9% and V3.8% respectively. The mean interday variability (coefficient of variation) of QLNC120 and lapatinib QC samples were V7.6% andV9.8% respectively. No interferences were observed in blank plasma or plasma spiked with internal standard.

The PK data was analyzed by using Phoenix WinNonlin Software (version: Phoenix WinNonlin 6.3). Non-compartmental analysis (NCA) was used to calculate the area under the plasma concentration-time curve over the time interval from 0 to 24h (AUC_0–24_, ss), maximum measured concentration (C_max_), and terminal half-life (t_1/2_).

### Targeted mutation next generation sequencing

According to the manufacturer's instructions, genomic DNA was isolated from 5-10 slides of 10 μm FFPE (formalin-fixed, paraffin-embedded) samples with high estimated tumor content (>30% tumor nuclei)(TIANGEN Biotech, Beijing, China). The amount of DNA for each patient was quantified by using the Qubit 2.0 fluorometer (Life Technologies, Foster City, CA) (50-100 ng/μl). DNA libraries were generated from 10 ng of DNA per sample by using the Ion AmpliSeq Cancer Hotspot v2 Panel (Life Technologies, Foster City, CA) and the Ion Ampliseq library kit 2.0 (Life Technologies, Foster City, CA) according to manufacturer's instructions. The Panel targets more than 700 mutational hotspot regions to detect mutations in 50 tumor suppressor genes and proto-oncogenes. Sequencing of multiplexed templates was performed by using the IonTorrent Personal Genome Machine (Life Technologies, Foster City, CA) on Ion 316 chips followed manufacturer's instructions. The initial data was processed with Ion Torrent software Torrent Suite (version 5.0) to generate sequence reads, trim adapter sequences and filter poor signal reads. Variant Caller plugin (version 3.6.63335) and Coverage analysis plugin (version 4.0) were applied to detect variants and analyze coverage and sequencing depth respectively. Prioritized variants were compared to known somatic variants reported in the Pubmed database.

### Statistical methods

The association between HRAS mutation and PFS was evaluated using Cox regression analysis. Risks were reported as hazards ratios (HR) along with their 95% confidence interval (CI). Furthermore, overall survival curves, estimated by Kaplan-Meier method, were compared using the log-rank test in SPSS 20.0. The differences were considered statistically significant when p < 0.05.
